# Body Mass Index and Cardiovascular Risk Markers: A Large Population Analysis

**DOI:** 10.3390/nu17050740

**Published:** 2025-02-20

**Authors:** Bela F. Asztalos, Giuseppina Russo, Lihong He, Margaret R. Diffenderfer

**Affiliations:** 1 Boston Heart Diagnostics, 200 Crossing Blvd, Suite 200, Framingham, MA 01702, USA; lhe@diatherix.eurofinsus.com (L.H.); margaret.diffenderfer@bostonheart.eurofinsus.com (M.R.D.); 2 Department of Clinical and Experimental Medicine, University of Messina, 98122 Messina, Italy; giuseppina.russo@unime.it

**Keywords:** obesity, ASCVD, lipids, lipoproteins, lipoprotein particles, fatty acids, cholesterol production, cholesterol absorption, inflammation

## Abstract

**Background/Objectives.** An elevated body mass index (BMI) has been added to the new American Heart Association atherosclerotic cardiovascular disease (ASCVD) risk model. Our goal in this study was to examine the relationships between BMI and traditional and non-traditional ASCVD risk factors. **Methods.** We measured levels of blood glucose, insulin, lipids, lipoproteins, sterols, fatty acids, markers of inflammation and oxidative stress, and hormones in 226,000 middle-aged and elderly subjects (55% women) and associated those parameters to BMI in 5 groups (BMI 20–25, 25.1–30, 30.1–35, 35.1–40, and >40 kg/m^2^). **Results**. BMI and age were inversely correlated in both sexes. All of the traditional and non-traditional ASCVD risk markers, except low-density lipoprotein cholesterol (LDL-C), changed significantly in unfavorable ways in both sexes with increasing BMI. The largest changes were observed in the high sensitivity C-reactive protein, which increased 6- and 8-fold, and insulin, which increased 4- and 3-fold between the lowest and highest BMI groups in men and women, respectively. Although the LDL-C levels changed little, small dense LDL-C and triglyceride levels increased significantly with increasing BMI. Markers of cholesterol synthesis were positively associated with BMI, while markers of cholesterol absorption and omega-3 fatty acids were inversely associated with BMI. Concentrations of high-density lipoprotein cholesterol (HDL-C) and the athero-protective, large-size HDL particles were also inversely associated with BMI. Our analysis indicated that the associations between an elevated BMI and unfavorable changes in major ASCVD risk factors were independent of age in both sexes. Moreover, we observed that ASCVD risk factors started changing unfavorably with increasing BMI even in the normal weight range (BMI 20–25 kg/m^2^). **Conclusions.** An elevated BMI is associated with unfavorable changes in traditional and non-traditional ASCVD risk factors independent of age. Therefore, maintaining a normal BMI, preferably by an active lifestyle, and, if necessary, weight-managing medication, is very important to avoid developing conditions leading to ASCVD.

## 1. Introduction

Obesity is one of the greatest public health issues in the United States (US). It is estimated that only about one-thirdof the adult population has normal weight (BMI 20–25 kg/m^2^), while more than 40% are obese (BMI ≥ 30 kg/m^2^) [1,2]. Moreover, the number of morbidly obese subjects (BMI ≥ 40 kg/m^2^) has been increasing for decades [3]. Recent data indicate that mortality is significantly increased among obese people [4,5]. Obesity increases the risk of developing dyslipidemia (high triglyceride [TG] and small dense low-density lipoprotein cholesterol [sdLDL-C] levels and low high-density lipoprotein cholesterol [HDL-C] levels), type-2 diabetes, hypertension, and systemic inflammation, all of which are established risk factors for atherosclerotic cardiovascular disease (ASCVD).

Some large prospective analyses have indicated that the link between obesity and ASCVD is mediated largely by hypertension, dyslipidemia, and diabetes among other conditions [6]. However, other prospective studies have suggested that the risk for ASCVD is still high in obese people even after accounting for these risk factors [7,8]. Some studies reported that metabolically healthy obesity was not associated with incident myocardial infarction [9,10,11]. However, Sjostrom and colleagues reported that ASCVD risk improved significantly only after at least a 10 kg weight loss [12]. Losing weight and especially maintaining weight loss long term can be very challenging though.

A recent meta-analysis pooling data from 10 large US prospective cohorts found that obesity was associated with shorter longevity and significantly increased risk for getting ASCVD at an earlier age [13]. With the introduction of glucagon-like peptide-1 (GLP-1) receptor agonists, losing a significant amount of weight and maintaining weight loss on a long-term basis is now possible. However, the long-term side effects of these medications are unknown.

Although it is well recognized that ASCVD is the leading cause of death in both men and women in the US, ASCVD risk is profoundly different in the two sexes, with women showing a delay in ASCVD onset due to the protective effects of estrogen. Obesity is also a sex-dependent risk factor mainly because men and women differ in adipose tissue distribution and function [14]. Obesity is more prevalent in women than in men worldwide [15,16]. A sex-specific impact of different obesity/metabolic phenotypes on long-term ASCVD outcomes in patients having acute coronary syndrome has also been recently demonstrated [17]. These data suggest that obesity may differently affect cardio-metabolic risk factors and ASCVD risk in men and women.

In order to elucidate the role of obesity in ASCVD risk factors in both sexes, we measured traditional and emerging cardio-metabolic risk factors in a very large US population, divided the data by sex and BMI, and calculated differences among the groups. To our knowledge, such a comprehensive study including such a large population (226,000 subjects, 55% female) has not been reported before.

## 2. Materials and Methods

### 2.1. Study Population

The samples in these analyses were obtained from 101,982 male and 124,018 female subjects. The results were selected from a large pool of results from samples sent by healthcare providers throughout the US to Boston Heart Diagnostics, for the measurement of biomarkers of ASCVD risk in 2017–2018. Subjects were required to be fasted for at least 8 h prior to having their blood drawn; samples from subjects not fasting were excluded from the study population. Information on the subjects’ sex, age, BMI, and health status (history of ASCVD, diabetes, hypertension, and use of lipid-lowering medication) was collected from their healthcare providers. All laboratory data were de-identified prior to analysis. The study was determined to be exempted from institutional review board approval under 45 CFR46.104(d) by the Advarra Institutional Review Board (Columbia, MD, USA) on 25 September 2020.

### 2.2. Laboratory Measurements

Blood was collected and put on ice immediately and sent to Boston Heart Diagnostics on ice packages equipped with a temperature-monitoring device. Samples were acceptable for the analyses if the boxes arrived within 48 h after collection and the temperature in the box was <7 °C. All laboratory measurements were performed at Boston Heart Diagnostics.

Most assays were measured by automated standardized methods as previously described [18], using Roche COBAS automated analyzers with reagents obtained from Roche Diagnostics (Indianapolis, IN, USA) including total cholesterol, LDL-C, sdLDL-C, triglycerides, HDL-C, apolipoproteins (apo) A and B, Lp(a), glucose, insulin, and high sensitivity C-reactive protein (hsCRP). Hemoglobin A1c (HbA1c) was measured using a Sysmex NX9000 automated instrument (Norderstedt, Germany). Adiponectin, fibrinogen, lipoprotein-associated phospholipase A2 (LpPLA_2_), and myeloperoxidase (MPO) were measured using a DZ-lite c270 (Diazyme, Poway, CA, USA). LDL particle number (LDL-P) and size were measured using a nuclear magnetic resonance (NMR) spectroscopic instrument and Numares software version 1.0.1 (Regensburg, Germany). The within- and between-run coefficients of variation for automated assays were <4%. ApoA-I-containing HDL subpopulations were measured by 2-dimensional non-denaturing gel-electrophoresis, immunoblot, and image analysis, as described earlier [19]. Total, trans, saturated, monounsaturated, and polyunsaturated (arachidonic acid [AA], linoleic acid [LA], α-linolenic acid [ALA], docosahexaenoic acid [DHA], and eicosapentaenoic acid [EPA]) fatty acids, and markers of cholesterol absorption (β-sitosterol, campesterol, and cholestanol) and synthesis (desmosterol and lathosterol) were measured by gas chromatography–mass spectroscopy, as previously described [20,21]. The relative concentration of the sterols was calculated as μmol sterol × 100/mmol of total cholesterol. Non-HDL-C was calculated as total cholesterol—HDL-C. Very low-density lipoprotein cholesterol (VLDL-C) was calculated as total cholesterol—(direct LDL-C + HDL-C).

### 2.3. Statistical Analyses

Subjects were divided into 5 groups based on BMI: normal weight (BMI 20–25 kg/m^2^), overweight (BMI 25.1–30 kg/m^2^), moderately obese (BMI 30.1–35 kg/m^2^), very obese (BMI 35.1–40 kg/m^2^), and morbidly obese (BMI > 40 kg/m^2^). Subjects having BMI values < 20 kg/m^2^ were excluded from all analyses, and subjects having BMI > 40 kg/m^2^ were excluded from some analyses, as indicated. The descriptive data in Table 1, Table 2 and Table 3 are expressed as median values with the interquartile range (IQR) based on the 25th and 75th percentile values. Categorical variables are reported as frequencies and percentages. The trend of changes among the 5 BMI groups was calculated for each variable separately in the two sex groups and, in some cases, separately in the ASCVD and non-ASCVD groups. For some analyses, data were adjusted for age. Statistical differences were accepted at the *p* < 0.05 level. All statistical analyses were performed using R software version 4.4.2.

## 3. Results

In Table 1, we show the anthropometric parameters, lipids, and lipoproteins of male and female subjects, stratified into 5 groups by BMI: normal weight (BMI 20–25 kg/m^2^), overweight (BMI 25.1–30 kg/m^2^), moderately obese (BMI 30.1–35 kg/m^2^), very obese (BMI 35.1–40 kg/m^2^), and morbidly obese (BMI > 40 kg/m^2^). Only 16.8% of men and 28.7% of women were in the normal weight group. Age decreased with increasing BMI in both sexes. The prevalence of ASCVD increased modestly with increasing BMI in both sexes. Although systolic blood pressure increased by <10% between the normal weight and morbidly obese groups, the prevalence of hypertension was more than double in the morbidly obese group compared to the normal weight group in both sexes.

The use of lipid-lowering medications (lipid-Rxs) progressively increased from the normal weight to the very obese group without further increment in the morbidly obese group of either sex. Generally, women were less likely to be on lipid-Rx than men. The total cholesterol and LDL-C levels changed very little among the five BMI groups in both men and women. The absolute and relative concentration of sdLDL-C, and the concentration of LDL-P, TG, VLDL-C, non-HDL-C, and apoB peaked in the moderately obese and very obese groups. The concentration of Lp(a) did not change with increasing BMI in the men, but it did increase in the women groups. The concentrations of HDL-C, apoA-I, and apoA-I in very large α-1 HDL, large α-2 HDL, and very small preβ-1 HDL were inversely associated with BMI in both sexes. All HDL-related parameters were higher in the female groups compared to male groups with similar BMI values.

**Figure 1:**
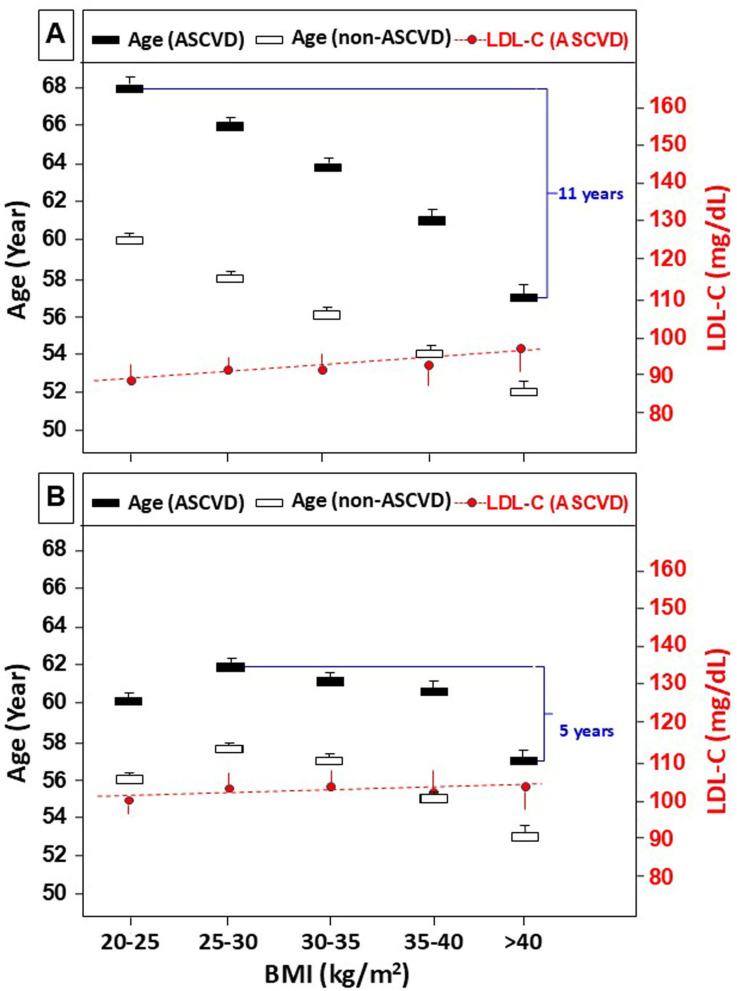
Age and LDL-C levels of subjects with or without ASCVD, grouped by BMI. Panel (**A**), men; panel (**B**), women. Subjects were divided into 5 BMI categories: 20–25 kg/m^2^; 25.1–30 kg/m^2^; 30.1–35 kg/m^2^; 35.1–40 kg/m^2^; >40 kg/m^2^. Data points represent median level +/− standard error. Age was significantly different between consecutive BMI groups in both sexes. Abbreviations: ASCVD, atherosclerotic cardiovascular disease; BMI, body mass index; LDL-C, low-density lipoprotein cholesterol. The figures demonstrate that in this population, ASCVD women were significantly younger than men in each BMI group and that BMI was inversely associated with age in the ASCVD and non-ASCVD groups in both sexes.

In Figure 1, we show the median age in the five BMI groups separately in ASCVD and non-ASCVD male and female subjects. Normal weight ASCVD-positive men were 8 years older than their non-ASCVD counterparts. Among ASCVD-positive normal weight and morbidly obese men, age decreased by 11 years and the LDL-C level increased by 16 mg/dL. Among non-ASCVD normal weight and morbidly obese men, age also decreased, but only by 8 years. Normal weight ASCVD-positive women were 8 years younger compared to normal weight ASCVD-positive men. From the normal weight group, age increased by 2 years in the overweight group, then it gradually decreased until it was 5 years lower in the morbidly obese group. Among ASCVD-positive women, LDL-C increased by only 4 mg/dL between the normal weight and the morbidly obese groups.

Table 2 shows markers of glucose homeostasis and inflammation and oxidative stress in the five male and female groups. The prevalence of diabetes and pre-diabetes and the levels of insulin and insulin-resistance increased several-fold between the normal weight and morbidly obese groups in both sexes. Fasting blood glucose and HbA1c also increased, although moderately, with increasing BMI. Adiponectin levels were higher in each BMI group of women compared to men, and decreased with BMI in both sexes. The hsCRP greatly increased and MPO increased, while LpPLA_2_ decreased, with increasing BMI, with similar trends in both men and women.

Data on cholesterol homeostasis markers and fatty acids are shown in Table 3. The relative concentrations of cholesterol synthesis markers (lathosterol and desmosterol) increased, while the relative concentrations of cholesterol absorption markers (β-sitosterol, campesterol, and cholestanol) decreased with increasing BMI in both men and women. Concentrations of plasma total, trans, saturated, and monounsaturated fatty acids increased, while concentrations of plasma omega-3 (DHA and EPA) and omega-6 (LA) polyunsaturated fatty acids decreased with increasing BMI in both sexes.

**Figure 2:**
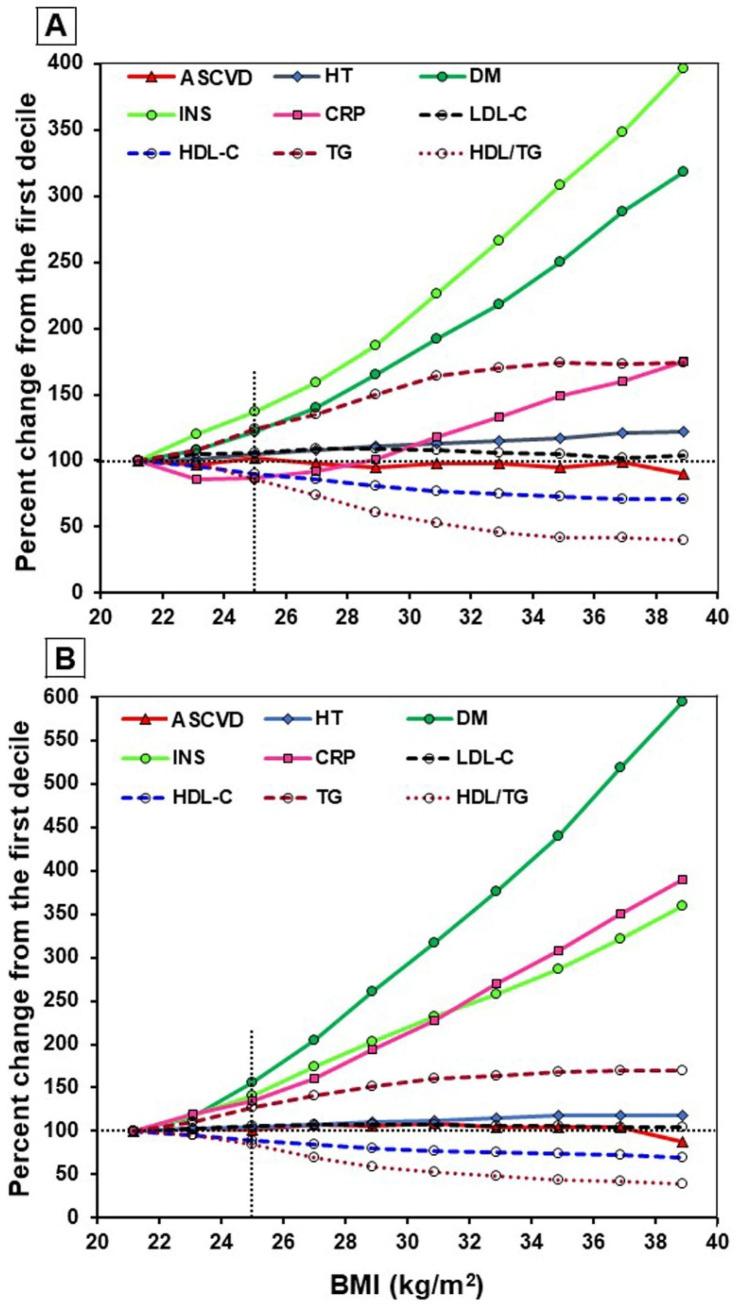
Scatter plot of ASCVD risk markers versus BMI. Panel (**A**), men (n = 101,982); panel (**B**), women (n = 124,018). Subjects having BMI 20–40 kg/m^2^ were stratified into 10 groups by 2-unit intervals of BMI. All variables were age-adjusted. Median levels were calculated and percent changes from the first decile were plotted against the median BMI of each interval. Abbreviations: ASCVD, atherosclerotic cardiovascular disease; BMI, body mass index; CRP, C-reactive protein; DM, diabetes mellitus; HDL-C, high-density lipoprotein cholesterol; HT, hypertension; INS, insulin; LDL-C, low-density lipoprotein cholesterol; TGs, triglycerides. The plots demonstrate the differences in the associations between BMI and major ASCVD risk factors in men and women after adjusting data for age.

Because BMI and ASCVD risk factors were strongly correlated with age, we carried out age-adjusted analyses. Subjects were stratified into 10 groups by 2 units of BMI between 20 and 40 kg/m^2^. The calculated percent changes from the first decile for each variable were then plotted as data points versus BMI for each group (Figure 2). The prevalence of ASCVD changed very little in the first 9 deciles, but was lower by 10% (men) and 13% (women) in the highest BMI group (median BMI ≥ 39 kg/m^2^). Diabetes, insulin, and hsCRP increased several-fold between the lowest and the highest BMI groups in both sexes. Increases in diabetes and hsCRP were about twice as high in women as compared to men. TG increased significantly in each of the first six deciles and then the curve flattened in both sexes. Compared to the first decile, HDL-C decreased continually and was about 30% lower in the last decile in both sexes.

## 4. Discussion

Although several studies have documented that obesity is significantly associated with increased risk for ASCVD, the direct role of obesity in ASCVD risk is still debated [22,23]. In this study, we explored the associations between BMI and several conventional and non-conventional ASCVD risk markers (lipids, lipoproteins, glucose homeostasis, inflammation, and oxidative stress) in fasting samples sent for testing from 226,000 US subjects. We also explored whether the associations between BMI and common ASCVD risk factors were sex-specific, as reported for diabetes, one of the most common disorders in obese subjects [24,25,26]. In a recent review, Huebschmann et al. emphasized the lack of research on sex differences in examining the complex role of diabetes in ASCVD risk [27]. Men and women differ in fat metabolism, its deposition patterns, and the health consequences of obesity [28]. In a Swedish study, the metabolically healthy obesity phenotype was associated with an increased ASCVD risk only in men [29], whereas in the San Antonio Heart Study, no sex differences were noted [30]. Sex differences in the long-term ASCVD outcomes of patients according to different obesity phenotypes have been recently reported, with obesity per se being a stronger risk factor in men than in women [17].

Similarly to others [1,2], we have found that only a minority of men and women had normal weight (BMI 20–25 kg/m^2^; 16.8% and 28.7%, respectively), while most subjects fell into the overweight group (BMI 25–30 kg/m^2^; 40.7% and 29.4%, respectively) and more than 15% of subjects had a BMI > 35 kg/m^2^ in both sexes (Table 1). Shockingly, 5.6% of men and 7.8% of women had a BMI > 40 kg/m^2^ and quite a large number of subjects had a BMI > 50 kg/m^2^ in both sex groups. In agreement with others, we found that women had lower rates of ASCVD in each BMI category compared to men [31]. In both sex groups, ASCVD increased significantly, although only slightly, with increasing BMI. We have tested the association between BMI and age separately in ASCVD and non-ASCVD subjects (Figure 1). As expected, ASCVD-positive men were older (about 8 years) compared to BMI-matched non-ASCVD men. Moreover, age decreased by 2 years in each higher BMI strata, resulting in an 11-year gap between normal weight and morbidly obese men.

Unexpectedly, we have observed that the normal weight women group was 8 years younger than the corresponding men group. Among ASCVD-positive women, age first increased by 2 years from the normal to the overweight group, but from there it decreased in each higher BMI strata. The morbidly obese ASCVD-positive women were 5 years younger as compared to the overweight group. The age gap between ASCVD-positive women and men is in contrast with the observation by other investigators. One explanation could be that 2/3 of women in our pool were >51 years old, and therefore, were postmenopausal and lacking the known hormonal protection against ASCVD. Another explanation could be that in the last decade, the diagnosis for ASCVD and its risk factors has improved and shifted toward younger ages, especially among women [32]. Moreover, women are more likely than men to seek medical treatment; therefore, they may be diagnosed earlier. The declining age of the higher BMI-ASCVD-positive groups is probably due to increased mortality at a younger age because of a higher burden of comorbidities, such as atherogenic dyslipidemia, diabetes, hypertension, and inflammation [5,13,33]. The consequences are that obese people not only die earlier but also have a shorter disease-free lifetime.

The total cholesterol and LDL-C did not change much, but diabetes, insulin, and hsCRP increased several-fold with increasing BMI (Table 2), and that was true even after adjusting data for age (Figure 2). Insulin resistance and the accompanying hyperinsulinemia can directly affect lipid and lipoprotein metabolism, leading to the development of atherogenic dyslipidemia (low HDL-C; high TG). Generally, not all subjects with low HDL-C have high TG, but the vast majority of subjects with a high TG level have low HDL-C. Therefore, treating high TG concentrations definitively would improve dyslipidemia. The pathogenic role of hypertriglyceridemia in ASCVD risk is being increasingly recognized, and it is reported that elevated TG levels were associated with increased ASCVD risk, even at levels considered “normal” (<150 mg/dL) [34].

A high TG level is associated with a high concentration of sdLDL-C, which is the most atherogenic apoB-containing lipoprotein particle class [35]. A high BMI was also associated with increased TG concentration and an unfavorable HDL profile: low levels of HDL-C, apoA-I, and apoA-I in very large α-1 HDL and very small preβ-1 HDL. In contrast, normal weight healthy individuals have high levels of apoA-I in very large α-1 HDL and low levels of apoA-I in very small preβ-1 HDL [36,37]. It has been documented that very small preβ-1 discoidal HDL particles accept excess free cholesterol and phospholipid from macrophages in the vessel wall via the ATP-binding cassette transporter A1 (ABCA1) pathway, as well as from other cells throughout the body [38,39,40]. Thereafter, lecithin–cholesterol acyltransferase (LCAT) acts on these particles to allow for the formation of cholesteryl ester and large spherical α-migrating HDL particles. The large and very large α-2 and α-1 HDL particles then deliver cholesterol (both free and esterified) to the liver for excretion into bile via the scavenger receptor class B type 1 (SR-B1) receptor (reverse cholesterol transport). We believe that subjects having low levels of both preβ-1 HDL and α-1 HDL particles have compromised reverse cholesterol transport.

In order to better define lipid abnormalities in obese subjects, cholesterol synthesis (β-sitosterol, campesterol, and cholestanol) and absorption (lathosterol and desmosterol) markers and the concentrations of various fatty acids were also measured in plasma (Table 3). There was clear evidence that markers of cholesterol synthesis increased and markers of cholesterol absorption decreased with increasing BMI values in both men and women. These results were unexpected since the use of lipid-lowering medications (mainly statins) increased with increasing BMI. Statins are known to decrease markers of cholesterol synthesis and increase markers of cholesterol absorption. It should be noted that most subjects were not on statins. We assume that an elevated BMI has significant effects on cholesterol homeostasis.

In obese, insulin-resistant people, a high insulin level increases de novo fatty acid synthesis in the liver, resulting in a high concentration of TG, which upregulates apoB and cholesterol synthesis and secretion [41]. We found that free fatty acid concentration in plasma increased with increasing BMI, which might have contributed to increased TG production in the liver and insulin resistance in the muscle. Concentrations of trans, saturated, and monounsaturated fatty acids also increased, while concentrations of all polyunsaturated fatty acids decreased with increasing BMI. Most importantly, both DHA and EPA concentrations were about 25% and 35% lower, respectively, in the morbidly obese groups compared to the normal weight group in both genders. These data clearly indicate that obese people consumed a poorly balanced diet low in long-chain omega-3 fatty acids and high in saturated fatty acids.

An important observation was that the unfavorable changes in glucose, lipid, and lipoprotein homeostasis, and inflammation and oxidative stress started in the normal weight range (BMI 20–25 kg/m^2^).

The concentrations of liver and kidney function markers changed little and stayed in the normal range in all BMI groups in both sexes (Supplemental Table S1). The level of vitamin D was inversely associated with BMI and was about 30% lower in the morbidly obese compared to the normal weight group in both men and women. The role of vitamin D in ASCVD is not fully understood, but several articles have connected low vitamin D levels with increased risk for ASCVD [42,43]. Thyroid gland hormones changed very little and stayed in the normal range, but concentrations of sex hormones decreased substantially with increasing BMI within all BMI groups (Supplemental Table S2).

We agree with other reports [44,45] that metabolically healthy overweight is not a fully benign condition, because the obesity-associated abnormalities, such as dyslipidemia, insulin resistance, diabetes, hypertension, and systemic inflammation, appear with time, and inevitably have a deleterious effect on health in obese people. Therefore, “healthy obesity” is not permanent but rather a transitory state with ASCVD risk increasing over time as compared to lean healthy subjects [46,47,48]. Ideally, weight reduction and maintenance should be the solution for decreasing ASCVD risk and comorbid conditions in obese subjects, as indicated by a recent publication documenting that weight loss significantly reduced inflammation and favorably changed CVD risk factors, including HDL-C ratios, particularly TC/HDL-C, LDL-C/HDL-C, and apo A-1 [49]. However, in real life, very few people are able to lose an adequate amount of weight, especially for long term. The new weight loss drugs (GLP-1 receptor agonists) appear to be rapidly changing the way obesity and its comorbid conditions are being treated.

This study has many strengths and limitations that should be taken into account when interpreting the results. The major weakness of the study is that histories of ASCVD and diabetes were self-reported and we did not have information on the duration of either condition. It is well known that self-reported data are not very accurate. Having a detailed medical history of ASCVD, diabetes, and drug use for those conditions would highly strengthen the results. Lipid-lowering medications were also self-reported. Historically self-reported data are not very reliable. Information about other medications and smoking status were also lacking for most subjects. Another important weakness is that we did not have data on waist circumference; therefore, we could not adjust for type of obesity. There was no information on the ethnicity of the patients; however, the large number of subjects and the distribution of the doctors’ offices sending samples to us were from all over the US, suggesting that our population was ethnically diverse. Information about the menopausal status in females was not collected, but 2/3 of the women were ≥51-year-olds, which indicates that the majority were post-menopausal.

The major strength of our study is the very large sample size and the measurements of several traditional and non-traditional ASCVD risk markers, which are not widely available. Moreover, all measurements were done in our laboratory. This is a very important factor, because different laboratory practices and conditions for sample collection, handling, and storage significantly affect the outcome of laboratory measurements. There are numerous publications about the associations of BMI with ASCVD risk, but many of the large-scale studies are meta-analyses using measurements of several laboratories. In our case, blood collection, shipping, and handling of samples were well controlled and all measurements were accomplished within 72 h after blood collection in the same laboratory.

## 5. Conclusions

Although the prevalence of ASCVD changed only modestly with increasing BMI, the prevalence of major ASCVD risk factors including hypertension, insulin resistance, diabetes, dyslipidemia, and markers of inflammation significantly increased in each increasing BMI group in both sexes. Unexpectedly, ASCVD risk factors started changing unfavorably with increasing BMI even in the normal weight range (BMI 20–25 kg/m^2^). Moreover, BMI was inversely associated with age in all subjects, and in ASCVD-positive and non-ASCVD subjects in both sexes. An elevated BMI was associated with an unhealthy HDL profile (low levels of HDL-C, apoA-1, and apoA-I in very large α-1 HDL and very small preβ-1 HDL particles), indicating compromised reverse cholesterol transport. Compared to normal weight subjects, each higher BMI category was associated with a shorter ASCVD-free life span: about 2 years in men and about 1 year in women. An increased BMI had similar effects on all measured parameters in both sexes. Considering the strong positive correlations between BMI and several ASCVD risk factors and comorbid conditions, we do not support the concept of metabolically healthy obesity. Maintaining a normal BMI, preferably by an active lifestyle and, if necessary, weight-managing medication, is very important to avoid developing conditions leading to ASCVD.

## Figures and Tables

**Table 1:** Anthropometric parameters, lipids, and lipoproteins in men (n = 101,982) and women (n = 124,018) *.

	BMI Cut Points					*p* (Trend) ^†^
	20–25	25.1–30	30.1–35	35.1–40	>40	
Men						
Percent of total	16.8	40.7	25.6	9.9	5.6	
BMI (kg/m^2^)	23 (24–21)	27.1 (28–26)	32 (33–31)	37 (38–36)	44 (48–42)	NA
Age (year)	56 (66–44)	58 (67–47)	56 (66–46)	55 (64–45)	51 (62–42)	<0.001
ASCVD (%)	9.7	9.7	9.8	10.2	10.2	<0.001
sBP (mmHg)	122 (130–120)	126 (140–120)	130 (140–120)	130 (140–120)	132 (140–120)	<0.001
Hypertension (%)	17.6	23.4	30.0	35.1	38.3	<0.001
Lipid-Rx (%) ^‡^	17.1	21.0	22.8	22.9	20.6	<0.001
TC (mg/dL)	180 (210–150)	183 (210–150)	181 (210–150)	178 (210–150)	175 (200–150)	<0.001
LDL-C (mg/dL)	109 (140–84)	114 (140–87)	113 (140–86)	112 (140–87)	110 (140–86)	<0.001
sdLDL-C (mg/dL)	23 (32–17)	27 (40–19)	30 (44–21)	30 (44–21)	28 (42–20)	<0.001
sdLDL-C (%)	21 (27–18)	24 (31–19)	27 (35–21)	27 (36–21)	26 (35–20)	<0.001
LDL-P (nmol/L)	1160 (1500–850)	1290 (1700–960)	1330 (1700–990)	1330 (1700–1000)	1310 (1700–1000)	<0.001
TG (mg/dL)	87 (120–64)	109 (160–78)	131 (190–93)	141 (200–100)	136 (200–99)	<0.001
VLDL-C (mg/dL)	13 (19–8)	16 (23–11)	19 (27–13)	21 (29–15)	21 (29–15)	<0.001
nonHDL (mg/dL)	128 (150–96)	133 (160–100)	136 (170–110)	136 (170–110)	134 (160–110)	<0.001
apoB (mg/dL)	89 (110–72)	95 (120–77)	97 (120–78)	97 (120–78)	96 (120–78)	<0.001
Lp(a) (mg/dL)	15 (41–15)	15 (40–15)	15 (36–15)	15 (35–15)	15 (37–15)	<0.001
HDL-C (mg/dL)	54 (65–45)	48 (58–40)	43 (51–36)	41 (48–35)	40 (47–34)	<0.001
apoA-I (mg/dL)	150 (170–130)	144 (160–130)	137 (150–120)	134 (150–120)	132 (150–120)	<0.001
α-1 (mg/dL)	28.8 (39–21)	23.1 (31–17)	19.2 (26–14)	17.4 (24–13)	16.7 (22–12)	<0.001
α-2 (mg/dL)	60.5 (70–53)	58.4 (68–51)	55.9 (64–48)	54.4 (63–47)	53.6 (62–46)	<0.001
α-3 (mg/dL)	21.9 (25–19)	23.1 (27–20)	24.2 (28–21)	24.6 (28–21)	24.3 (28–21)	<0.001
α-4 (mg/dL)	16.6 (20–14)	16.6 (20–14)	16.9 (20–14)	17.1 (20–14)	17.2 (20–14)	<0.001
preβ-1 (mg/dL)	8.8 (12–6)	8.8 (12–6)	8.5 (12–6)	8.2 (11–6)	7.8 (11–5)	<0.001
Women						
Percent of total	28.7	29.4	18.9	9.9	7.8	
BMI (kg/m^2^)	22.7 (24–21)	27.1 (28–26)	32 (33–31)	37 (38–36)	44 (48–42)	NA
Age (year)	56 (66–44)	58 (67–47)	56 (66–46)	55 (64–45)	52 (62–42)	<0.001
ASCVD (%)	5.4	6.6	6.5	6.1	6.5	<0.001
sBP (mmhg)	119 (130–110)	122 (130–110)	126 (140–120)	128 (140–120)	130 (140–120)	<0.001
Hypertension (%)	13.0	29.9	25.8	28.7	32.2	<0.001
Lipid Rx ^‡^	10.2	13.6	14.6	14.9	13.5	<0.001
TC (mg/dL)	200 (230–170)	201 (230–170)	197 (230–170)	193 (220–170)	187 (220–160)	<0.001
LDL-C (mg/dL)	115 (140–92)	121 (150–96)	121 (150–97)	120 (150–96)	117 (140–94)	<0.001
sdLDL-C (mg/dL)	23 (31–18)	26 (36–19)	27 (38–20)	27 (38–20)	26 (37–19)	<0.001
sdLDL-C (%)	20 (24–17)	21 (27–18)	22 (29–19)	23 (29–19)	22 (29–18)	<0.001
LDL-P (nmol/L)	1180 (1500–900)	1320 (1700–1000)	1380 (1700–1100)	1380 (1700–1100)	1360 (1700–1100)	<0.001
TG (mg/dL)	80 (110–60)	103 (140–75)	118 (170–85)	125 (170–91)	125 (170–91)	<0.001
VLDL-C (mg/dL)	11 (17–7)	15 (22–10)	17 (24–12)	18 (25–13)	18 (25–13)	<0.001
nonHDL (mg/dL)	127 (160–100)	137 (170–110)	140 (170–110)	139 (170–110)	136 (160–110)	<0.001
apoB (mg/dL)	91 (110–76)	97 (120–80)	99 (120–82)	99 (120–82)	97 (120–80)	<0.001
Lp(a) (mg/dL)	15 (42–15)	16 (48–15)	17 (52–15)	17 (53–15)	19 (55–15)	<0.001
HDL-C (mg/dL)	70 (53–58)	61 (73–50)	55 (65–46)	52 (62–43)	49(59–42)	<0.001
apoA-I (mg/dL)	178 (200–160)	168 (190–150)	160 (180–140)	155 (170–140)	151 (170–130)	<0.001
α-1 (mg/dL)	41.5 (53–31)	33.4 (44–25)	28.9 (38–22)	26.5 (35–20)	24.9 (33–18)	<0.001
α-2 (mg/dL)	72.8 (83–63)	69.6 (80–60)	67.3 (77–58)	65.3 (75–56)	63.3 (73–55)	<0.001
α-3 (mg/dL)	22.4 (26–20)	23.4 (27–20)	23.9 (27–21)	23.9 (28–21)	23.5 (27–20)	<0.001
α-4 (mg/dL)	16.6 (20–13)	16.8 (20–13)	17.1 (21–14)	17.1 (21–14)	17.0 (21–14)	<0.001
preβ-1(mg/dL)	9.5 (13–7)	9.3 (13–6)	9.0 (13–6)	8.7 (12–6)	8.4 (12–5)	<0.001

* Data are median (Q3–Q1), except where percent (%) is indicated. ^†^ *p* (trend) was calculated from the normal weight (BMI 20–25 kg/m^2^) to the morbidly obese (BMI > 40 kg/m^2^) group. Significance level was accepted <0.05. ^‡^ Lipid Rx included use of statins, niacin, and fibrates. Apo, apolipoprotein; ASCVD, atherosclerotic cardiovascular disease; BMI, body mass index; HDL-C, high-density lipoprotein cholesterol; LDL-C, low-density lipoprotein cholesterol; LDL-P, low-density lipoprotein particle number; lipid-Rx, lipid-lowering medication; Lp(a), lipoprotein(a); sBP, systolic blood pressure; sdLDL-C, small dense low-density lipoprotein cholesterol; TC, total cholesterol; TG, triglycerides; VLDL-C, very low-density lipoprotein cholesterol.

**Table 2:** Markers of glucose homeostasis and inflammatory and oxidative stress in men and women *.

	BMI Cut Points					*p* (Trend) ^†^
	20–25	25.1–30	30.1–35	35.1–40	>40	
Men						
Glucose homeostasis markers						
T2DM (%)	9.8	13.5	20.8	28.0	35.5	<0.001
preDM	2.3 (5.5–0.9)	3.8 (10–1.6)	5.9 (17–2.4)	9.2 (24–3.5)	12 (33–3.8)	<0.001
Glucose (mg/dL)	95 (100–89)	98 (110–91)	101 (110–93)	103 (120–94)	104 (120–95)	<0.001
HbA1c (%)	5.5 (5.7–5.2)	5.5 (5.8–5.3)	5.7 (6.1–5.4)	5.8 (6.3–5.5)	5.9 (6.7–5.5)	<0.001
Insulin (U/L)	6.0 (9.6–4.0)	9.9 (14–6.1)	14 (21–10)	20 (29–13)	25 (38–16)	<0.001
HOMA-IR	1.5 (2.3–1.0)	2.3 (3.6–1.5)	3.7 (5.8–2.4)	5.3 (8.3–3.4)	6.8 (11–4.2)	<0.001
Adiponectin (μg/dL)	11.8 (17–8.4)	9.5 (13–6.8)	8.2 (11–6.0)	7.7 (10–5.6)	7.5 (10–5.6)	<0.001
Inflammatory and oxidative stress markers						
C-reactive protein (mg/L)	07 (1.6–0.3)	1.0 (2.2–0.5)	1.6 (3.2–0.8)	2.4 (4.6–1.2)	4.0 (7.3–2.0)	<0.001
Fibrinogen (mg/dL)	338 (400–290)	364 (420–320)	394 (460–340)	424 (500–370)	457 (530–400)	<0.001
LpPLA_2_ (ng/mL)	184 (220–150)	177 (210–150)	173 (200–140)	171 (200–140)	172 (200–140)	<0.001
MPO (pmol/L)	243 (320–180)	252 (330–200)	270 (350–210)	292 (380–230)	330 (420–260)	<0.001
Women						
Glucose homeostasis markers						
T2DM (%)	5.0	10.0	16.7	21.8	28.8	<0.001
preDM	0.9 (2.9–0.3)	1.8 (5.0–0.7)	3.0 (8.4–1.0)	4.2 (13.0–1.4)	6.8 (22.0–2.0)	<0.001
Glucose (mg/dL)	90 (97–85)	94 (100–87)	96 (110–89)	98 (110–90)	100 (110–91)	<0.001
HbA1c (%)	5.4 (5.6–5.2)	5.5 (5.8–5.3)	5.6 (6.0–5.4)	5.7 (6.1–5.4)	5.8 (6.3–5.5)	<0.001
Insulin (U/L)	6.3 (9.1–4.0)	9.2 (12.9–6.1)	13 (19–9)	16 (24–11)	20 (30–13)	<0.001
HOMA-IR	1.4 (2–0.9)	2.1 (3.2–1.4)	3.1 (4.8–2.0)	4.1 (6.4–2.6)	5.1 (8.3–3.3)	<0.001
Adiponectin (μg/dL)	17.3 (23–13)	14.2 (19–10)	11.9 (16–8.6)	10.8 (15–7.7)	9.9 (14–7.3)	<0.001
Inflammatory and oxidative stress markers						
C-reactive protein (mg/L)	0.8 (1.7–0.4)	1.5 (3.3–0.7)	2.8 (5.3–1.3)	4.1 (7.5–2.1)	6.4 (11–3.4)	<0.001
Fibrinogen (mg/dL)	337 (390–290)	374 (430–330)	406 (460–360)	436 (500–380)	467 (530–410)	<0.001
LpPLA2 (ng/mL)	187 (220–160)	181 (210–150)	176 (210–150)	172 (200–140)	169 (200–140)	<0.001
MPO (pmol/L)	247 (320–190)	275 (360–210)	301 (390–230)	325 (420–250)	362 (470–280)	<0.001

* Data are mean (Q3–Q1), except where percent is indicated. ^†^ *p* (trend) was calculated from the normal weight (BMI 20–25 kg/m^2^) to the morbidly obese (BMI > 40 kg/m^2^) group. Significance level was accepted <0.05. HbA1c, hemoglobin A1C; HOMA-IR, homeostatic model assessment for insulin resistance; LpPLA_2_, lipoprotein-associated phospholipase A2; MPO, myeloperoxidase; preDM, prediabetes; T2DM, type 2 diabetes mellitus.

**Table 3:** Markers of cholesterol absorption and synthesis and fatty acids in plasma in men and women *.

	BMI Cut Points					*p* (Trend) ^†^
	20–25	25.1–30	30.1–35	35.1–40	>40	
Men						
Cholesterol synthesis markers, normalized to total cholesterol ^‡^						
Lathosterol	96 (140–67)	107 (150–71)	119 (170–76)	130 (190–82)	142 (200–90)	<0.001
Desmosterol	68 (81–58)	71 (84–60)	73 (88–61)	75 (91–62)	75 (92–62)	<0.001
Cholesterol absorption markers, normalized to total cholesterol ^‡^						
β-sitosterol	158 (210–120)	137 (190–100)	122 (170–90)	112 (160–82)	103 (140–76)	<0.001
Campesterol	208 (280–150)	188 (260–140)	174 (240–120)	164 (230–120)	154 (210–110)	<0.001
Cholestanol	119(150–94)	111 (140–88)	105 (130–83)	103 (130–82)	101 (130–82)	<0.001
Fatty acids						
Total FA (μg/mL)	2980 (3400–2600)	3160 (3700–2700)	3300 (3900–2800)	3330 (4000–2800)	3250 (3900–2800)	<0.001
Trans FA (%)	0.46 (0.55–0.39)	0.47 (0.57–040)	0.49 (0.59–0.41)	0.50 (0.60–0.41)	0.49 (0.61–41)	<0.001
Saturated FA (%)	31 (32–29)	31 (33–30)	32 (34–30)	32 (34–31)	33 (34–31)	<0.001
MUFA (%)	21 (23–19)	22 (24–20)	22 (25–20)	23 (25–20)	23 (25–20)	<0.001
Polyunsaturated fatty acids						
Omega-6 FA (%)	43 (46–40)	42 (45–39)	41 (44–38)	40 (44–37)	41 (44–38)	<0.001
AA (%)	8.6 (9.9–7.2)	8.6 (10–7.2)	8.4 (10–7.0)	8.4 (9.9–7.0)	8.4 (9.9–7.0)	<0.001
LA (%)	32 (35–29)	31 (34–28)	30 (33–27)	30 (33–27)	30 (33–27)	<0.001
Omega-3 FA (%)	2.9 (4.1–2.1)	2.6 (3.7–2.0)	2.4 (3.2–1.9)	2.2 (3.0–1.8)	2.1 (2.7–1.7)	<0.001
ALA (%)	0.60 (0.75–0.48)	0.61 (0.76–0.49)	0.62 (0.77–0.50)	0.61 (0.77–0.50)	0.60 (0.73–0.50)	<0.001
DHA (%)	2.1 (2.8–1.5)	1.9 (2.6–1.5)	1.8 (2.3–1.3)	1.6 (2.1–1.3)	1.5 (2.0–1.2)	<0.001
EPA (%)	0.74 (1.3–0.54)	0.71 (1.1–0.51)	0.64 (0.94–0.47)	0.60 (0.86–0.45)	0.57 (0.75–0.43)	<0.001
Women						
Cholesterol synthesis markers, normalized to total cholesterol ^‡^						
Lathosterol	95 (130–69)	108 (150–74)	123 (170–82)	135 (190–89)	147 (200–97)	<0.001
Desmosterol	60 (71–52)	61 (73–52)	63 (76–53)	64 (77–54)	65 (79–55)	<0.001
Cholesterol absorption markers, normalized to total cholesterol ^‡^						
β-sitosterol	148 (200–110)	125 (170–91)	112 (150–82)	104 (140–76)	97 (140–71)	<0.001
Campesterol	188 (250–140)	167 (230–120)	154 (210–110)	144 (210–110)	141 (200–100)	<0.001
Cholestanol	113 (140–90)	105 (130–83)	101 (130–79)	99 (130–78)	100 (120–79)	<0.001
Fatty acids						
Total FA (μg/mL)	3190 (3600–2800)	3330 (3800–2900)	3380 (3900–2900)	3380 (3900–2900)	3310 (3900–2900)	<0.001
Trans FA (%)	0.42 (0.49–0.36)	0.43 (0.52–0.37)	0.44 (0.53–0.37)	0.45 (0.54–0.38)	0.45 (0.54–0.38)	<0.001
Sat FA (%)	31 (32–30)	31 (33–30)	32 (33–30)	32 (34–31)	32 (34–31)	<0.001
MUFA (%)	20 (22–18)	21 (23–19)	21 (24–19)	22 (24–20)	22 (24–20)	<0.001
Polyunsaturated fatty acids						
Omega-6 FA (%)	44 (46–41)	43 (46–40)	42 (45–39)	42 (45–39)	42 (45–39)	<0.001
AA (%)	8.6 (10–7.3)	8.7 (10–7.4)	8.7 (10–7.3)	8.6 (10–7.2)	8.6 (10–7.3)	<0.001
LA (%)	33 (36–30)	32 (35–29)	31 (34–29)	31 (34–28)	31 (33–28)	<0.001
Omega-3 FA (%)	3.1 (4.3–2.3)	2.7 (3.8–2.1)	2.5 (3.3–2.0)	2.3 (3.0–1.9)	2.2 (2.7–1.7)	<0.001
ALA (%)	0.60 (0.73–0.49)	0.61 (0.75–0.50)	0.62 (0.75–0.50)	0.61 (0.74–0.50)	0.60 (0.71–0.50)	<0.001
DHA (%)	2.3 (3.0–1.7)	2.0 (2.7–1.6)	1.8 (2.4–1.4)	1.7 (2.2–1.3)	1.6 (2.0–1.3)	<0.001
EPA (%)	0.77 (1.3–0.54)	0.70 (1.1–0.5)	0.64 (0.93–0.48)	0.61 (0.85–0.47)	0.57 (0.77–0.45)	<0.001

* Data are median (Q3-Q1). ^†^ *p* (trend) was calculated from the normal weight (BMI 20–25 kg/m^2^) to the morbidly obese (BMI > 40 kg/m^2^) group. Significance level was accepted <0.05. ^‡^ Normalized cholesterol absorption and synthesis markers are calculated as μmol sterol × 100/mmol of total cholesterol. AA, arachidonic acid; ALA, α-linolenic acid; DHA, docosahexaenoic acid; EPA, eicosapentaenoic acid; FAs, fatty acids; LA, linoleic acid; MUFAs, monounsaturated fatty acids.

## Data Availability

The data presented in this study are available on request from the corresponding author. Restrictions may apply due to legal reasons.

## Supplementary Materials

The following supporting information can be downloaded at https://www.mdpi.com/article/10.3390/nu17050740/s1, Supplemental Table S1. Markers of liver and kidney functions and homocysteine and vitamin D levels. Supplemental Table S2. Thyroid gland and sex hormones.

## Abbreviations

AA	arachidonic acid
ABCA1	ATP-binding cassette transporter A1
ALA	α-linolenic acid
Apo	apolipoprotein
ASCVD	atherosclerotic cardiovascular disease
BMI	body mass index
DHA	docosahexaenoic acid
EPA	eicosapentaenoic acid
GLP-1	glucagon-like peptide-1
HbA1c	hemoglobin A1c
HDL-C	high-density lipoprotein cholesterol
hsCRP	high sensitivity C-reactive protein
LA	linoleic acid
LCAT	lecithin cholesterol acyltransferase
LDL-C	low-density lipoprotein cholesterol
LDL-P	low-density lipoprotein particle number
Lipid-Rx	lipid-lowering medication
Lp(a)	lipoprotein(a)
LpPLA_2_	lipoprotein-associated phospholipase A2
MPO	myeloperoxidase
NMR	nuclear magnetic resonance
sdLDL-C	small dense low-density lipoprotein cholesterol
SR-B1	scavenger receptor class B type 1
TG	triglyceride
VLDL	very low-density lipoprotein cholesterol
