# Supplementary material for: Body Mass Index and Cardiovascular Risk Markers: A Large Population Analysis
Source: Nutrients. 2025 Feb 20;17(5):740. doi: 10.3390/nu17050740 (PMC11902009; doi:10.3390/nu17050740)
Supplement: Supplementary file 1 [file nutrients-17-00740-s001.zip › nutrients-3478531-supplementary.pdf]

Supplemental Table S1a. Markers of liver and kidney functions and homocysteine and vitamin D levels in men*

	BMI Cut Points					P (Trend)†
	20-25	25.1-30	30.1-35	35.1-40	>40	
Creatine kinase (U/L)	113 (70-80)	124 (190-87)	133 (200-91)	138 (220-94)	140 (230-92)	<0.001
Uric acid (mg/dL)	5.7 (6.6-5.0)	6.1 (7.0-5.3)	6.4 (74-5.5)	6.7 (7.6-5.7)	6.8 (7.8-5.8)	<0.001
Alkaline phosphatase (U/L)	66 (80-55)	67 (80-56)	68 (82-57)	70 (84-58)	73 (87-61)	<0.001
ALT (U/L)	20 (27-15)	23 (31-18)	26 (36-20)	29 (40-21)	29 (40-21)	<0.001
AST (U/L)	22 (27-18)	22 (27-19)	23 (28-19)	23 (30-19)	23 (30-19)	<0.001
BUN (mg/dL)	16 (19-13)	16 (19-13)	16 (19-13)	15 (19-13)	15 (18-12)	<0.001
Creatinine (mg/dL)	1.0 (1.1-0.9)	1.0 (1.2-0.9)	1.0 (1.2-0.9)	1.0 (1.1-0.9)	1.0 (1.1-0.8)	<0.001
eGFR (mL/min/1.73 m^2)	85 (97-72)	82 (94-70)	83 (95-70)	86 (99-72)	91 (100-76)	<0.001
GFRAA (mL/min/1.73 m^2)	98 (110-83)	95 (110-81)	97 (110-82)	99 (120-84)	105 (120-88)	<0.001
Homocysteine (μmol/L)	10 (13-8.7)	11 (13-9.0)	11 (13-9.0)	11 (13-9.0)	11 (13-8.8)	<0.001
Vitamin D (ng/mL)	35 (46-26)	32 (42-25)	29 (38-22)	27 (36-20)	25 (33-18)	<0.001

*Data are median (Q3-Q1).

†P (trend) was calculated from the normal weight (BMI 20-25 kg/m^2) to the morbidly obese (BMI>40 kg/m^2) group. Significance level was accepted <0.05.

ALT, alanine aminotransaminase; AST, aspartate aminotransferase; BUN, blood urea nitrogen; eGFR, estimated glomerular filtration rate; GFRAA, glomerular filtration rate in African Americans

Supplemental Table S1b. Markers of liver and kidney functions and homocysteine and vitamin D levels in women*

	BMI Cut Points					
	20-25	25.1-30	30.1-35	35.1-40	>40	P (Trend)†
Creatine kinase (U/L)	78 (110-58)	80 (110-59)	82 (120-60)	83 (120-60)	82 (120-58)	<0.001
Uric acid (mg/dL)	4.4 (5.1-3.7)	4.8 (5.7-4.1)	5.2 (6.1-4.5)	5.5 (6.5-4.7)	5.8 (6.8-4.9)	<0.001
Alkaline phosphatase (U/L)	62 (77-51)	70 (85-57)	75 (90-62)	78 (93-65)	80 (97-67)	<0.001
ALT (U/L)	15 (21-12)	17 (23-13)	18 (25-14)	19 (27-14)	19 (27-14)	<0.001
AST (U/L)	20 (24-17)	20 (24-17)	19 (24-16)	19 (24-16)	19 (24-15)	<0.001
BUN (mg/dL)	14 (17-11)	14 (17-11)	14 (17-11)	14 (17-11)	13 (17-11)	<0.001
Creatinine (mg/dL)	0.8 (0.9-0.7)	0.8 (0.9-0.7)	0.8 (0.9-0.7)	0.8 (0.9=0.7)	0.8 (0.9-0.7)	<0.001
eGFR (mL/min/1.73 m^2)	87 (99-75)	85 (97-71)	86 99-71)	87 (99-72)	90 (100-73)	<0.001
GFRAA (mL/min/1.73 m^2)	101 (120-87)	99 (110-83)	99 (110-83)	100 (120-83)	104 (120-85)	<0.001
Homocysteine (μmol/L)	8.4 (10-7.0)	8.8 (11-7.3)	9.0 (11-7.4)	9.0 (11-7.5)	9.2 (11-7.5)	<0.001
Vitamin D (ng/mL)	38 (50-29)	34 (46-26)	31 (42-23)	29 (30-21)	26 (36-18)	<0.001

*Data are median (Q3-Q1).

†P (trend) was calculated from the normal weight (BMI 20-25 kg/m^2) to the morbidly obese (BMI>40 kg/m^2) group. Significance level was accepted <0.05.

ALT, alanine aminotransaminase; AST, aspartate aminotransferase; BUN, blood urea nitrogen; eGFR, estimated glomerular filtration rate; GFRAA, glomerular filtration rate in African Americans

Supplemental Table S2a. Thyroid gland and sex hormones in men*

	BMI Cut Points					P (Trend)†
	20-25	25.1-30	30.1-35	35.1-40	>40	
TSH (μU/L)	1.9 (2.7-1.3)	1.9 (2.7-1.3)	1.9 (2.7-1.3)	1.9 (2.7-1.4)	1.9 (2.7-1.4)	<0.001
Free T3 (ng/dL)	3.1 (3.4-2.9)	3.1 (3.5-2.9)	3.3 (3.6-3.0)	3.3 (3.6-3.0)	3.3 (3.6-3.3)	<0.001
Free T4 (ng/dL)	1.3 (1.4-1.1)	1.3 (1.4-1.1	1.2 (1.4-1.1	1.2 (1.4-1.1	1.2 (1.3-1.1	<0.001
DHEAS (μg/dL)	181 (280-100)	164 (260-95)	156 (250-91)	149 (240-86)	116 (250-86)	<0.001
SHBG (nmol/L)	50 (68-36)	40 (57-29)	34 (46-24)	31 (43-22)	29 (40-21)	<0.001
Testosterone, total (ng/dL)	522 (670-400)	441 (580-330)	378 (500-280)	337 (440-250)	284 (390-200)	<0.001
Testosterone, free (pg/mL)	79 (100-62)	76 (95-60)	70 (89-54)	65 (82-50)	57 (77-42)	<0.001

*Data are median (Q3-Q1).

†P (trend) was calculated from the normal weight (BMI 20-25 kg/m^2) to the morbidly obese (BMI>40 kg/m^2) group.

Significance level was accepted <0.05.

DHEAS, dehydroepiandrosterone sulfate; SHBG, sex hormone binding globulin; TSH, thyroid stimulating hormone

Supplemental Table S2b. Thyroid gland and sex hormones in women*

	BMI Cut Points					P (Trend)†
	20-25	25.1-30	30.1-35	35.1-40	>40	
TSH (μU/L)	1.8 (2.7-1.2)	1.8 (2.7-1.2	1.8 (2.7-1.2	1.9 (2.8-1.2	2.0 (2.9-1.3)	<0.001
Free T3 (ng/dL)	2.9 (3.2-2.7)	3.0 (3.3-2.7)	3.0 (3.3-2.7)	3.0 (3.3-2.8)	3.0 (3.4-2.8)	<0.001
Free T4 (ng/dL)	1.2 (1.3-1.1)	1.2 (1.3-1.1)	1.2 (1.3-1.1)	1.2 (1.3-1.1)	1.2 (1.3-1.1)	<0.001
DHEAS (μg/dL)	120 (190-71)	111 (180-64)	108 (180-62)	109 (180-61)	108 (170-62)	<0.001
SHBG (nmol/L)	93 (130-67)	70 (100-48)	52 (75-37)	46 (66-33)	40 (60-28)	<0.001
Estradiol (pg/mL)	26 (87-25)	25 (69-25)	25 (63-25)	25 (64-25)	30 (67-25)	<0.001
Progesterone (ng/mL)	0.5 (1.8-0.3)	0.4 (1.0-0.2)	0.4 (0.8-0.2)	0.4 (0.7-0.2)	0.4 (0.7-0.2)	<0.001
LH (IU/L)	21 (38-6.6)	23 (37-7.5)	21 (34-7.1)	18 (31-6.7)	12 (25-6.0)	<0.001
FSH (IU/L)	30 (77-5.7)	40 (72-6.5)	35 (63-6.1)	25 (55-5.7)	11 (40-5.4)	<0.001
Testosterone, total (ng/dL)	17 (28-8.8)	17 (28-8.5)	17 (28-9.0)	19 (31-10)	21 (33-12)	<0.001
Testosterone, free (ng/dL)	1.7 (2.8-1.0)	2.0 (3.4-1.2)	2.6 (4.2-1.5)	3.0 (4.9-1.6)	3.5 (5.7-2.1)	<0.001

*Data are median (Q3-Q1).

†P (trend) was calculated from the normal weight (BMI 20-25 kg/m^2) to the morbidly obese (BMI>40 kg/m^2) group.

Significance level was accepted <0.05.

Measurement of levels of hormone E2 in the blood.

DHEAS, dehydroepiandrosterone sulfate; FSH, follicle-stimulating hormone; LH, luteinizing hormone; SHBG, sex hormone binding globulin; TSH, thyroid stimulating hormone
